# LUNAR: Full Moon or Eclipse? An exploration into tumor treating fields in lung cancer

**DOI:** 10.1016/j.tranon.2025.102397

**Published:** 2025-04-16

**Authors:** Timothée Olivier, Vinay Prasad

**Affiliations:** a Oncology Service, Geneva University Hospital, 4 Gabrielle-Perret-Gentil Street, 1205, Geneva, Switzerland; b Department of Epidemiology and Biostatistics, University of California San Francisco, 550 16th St, 2nd Fl, San Francisco, CA, 94158, USA

## Abstract

The LUNAR trial investigated the addition of Tumor Treating Fields (TTFs) to “standard therapy” in patients with metastatic lung cancer after at least one line of platinum-based chemotherapy. The “standard therapy” was either an anti-PD(L)1 therapy (immunotherapy) or docetaxel. The addition of TTFs provided a 3.3 months median survival gain. We raised concerns about LUNAR results internal and external validity.

First, patient selection and the control arm do not mirror current practice. Two-thirds of patients did not receive prior immunotherapy, which is standard in first-line treatment. Also, the “choice” of the “standard therapy” was restricted by drug availability, resulting in 41 % of patients not receiving immunotherapy during the trial – those allocated to receive docetaxel – had no prior exposure to immunotherapy. Some patients may have harbored actionable mutations, and did not receive targeted therapy.

Second, we raised statistical questions. The sample size was shrunk after an unplanned analysis, with unshared and unclear justifications. The decision may have been influenced by a chance deviation in data favoring the intervention. Also, as significantly more patients were censored after withdrawals in the TTFs group, informative censoring could have amplified the survival gain.

Third and last, without a sham-control design (the equivalent of placebo for devices), it's hard to isolate the impact of TTFs from the extra-attention associated with its administration (continuous 24/7 support, frequent home-based interactions).

Overall, LUNAR do not apply to clinical settings where immunotherapy and molecular testing is offered, and many factors may have artificially boosted the reported survival gain. A sham-controlled trial is needed to answer whether TTFs are beneficial.

## Highlights

• Tumor Treating Fields (TTFs) showed a survival gain in metastatic lung cancer.
• In the study, patient selection and the control arm did not mirror current practice.
• The subgroup who more closely approach the standard-of-care had no benefit.
• Unplanned sample size modification raises statistical concerns.
• Without a sham-control, TTFs were likely paired with additional supportive care.

## Graphical abstract

The images used in the graphical abstract are both from Tomruen, under CC BY-SA 4.0 :

**Figure:**
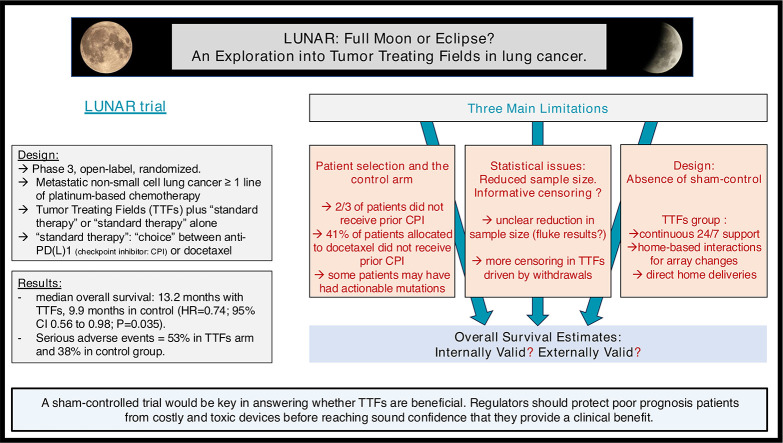

https://en.wikipedia.org/wiki/Full_moon#/media/File:Supermoon_Nov-14-2016-minneapolis.jpg, no changes were made.

https://en.wikipedia.org/wiki/Lunar_eclipse#/media/File:Lunar_eclipse_oct_8_2014_Minneapolis_4_46am.png, the image was cropped.

## Introduction

Tumor Treating Fields (TTFs) represent a novel medical device therapy with a unique mechanism of action [1]. Electric fields are delivered via a device connected to sticky "arrays" placed over the skin. In patients with metastatic non-small cell lung cancer (NSCLC), TTFs were investigated in the LUNAR trial, in addition to “standard therapy”, after at least one line of platinum-based chemotherapy [2]. The “standard therapy” was either an anti-PD(L)1 therapy (checkpoint inhibitors, CPI) or docetaxel chemotherapy. The addition of TTFs provided a 3.3 months median survival gain.

A survival benefit in such a setting would be a meaningful advance; however, we have reservations about the LUNAR's results. First, LUNAR has limited generalizability to current practice, as few patients resemble those enrolled in the study. Secondly, statistical questions surround the observed survival gain. The sample size was altered mid trial, and high rates of censoring in the survival analysis may have altered the results through informative censoring. Finally, without a sham-control, it's unclear whether the effects are due to the device itself or enhanced patient care provided only to patients treated with TTFs. We build the case that TTFs should be tested within a sham-controlled randomized trial, i.e. using a “placebo-device” in the control arm.

## The LUNAR trial

LUNAR randomized 276 patients who received either TTFs combined with “standard therapy” (*n* = 137) or “standard therapy” alone (*n* = 139). The standard therapy component was to be assigned to each patient by local investigators, and could be an anti-PD(L)1 therapy (nivolumab, pembrolizumab or atezolizumab), or docetaxel chemotherapy. The results showed a median overall survival of 13.2 months in the TTFs group compared to 9.9 months in the control group, indicating a difference of 3.3 months (hazard ratio, HR = 0.74; 95 % CI 0.56 to 0.98; *P* = 0.035). Serious adverse events were reported in 53 % of patients of the TTFs arm and 38 % of those in the standard therapy group.

## Patient selection, the “standard therapy” arm, and lack of biomarkers data

In patients with metastatic NSCLC without actionable mutations, first-line anti-PD(L)1 containing regimen are standard-of-care since years.

As a criteria to be enrolled in LUNAR, patients should have received prior platinum-based therapy, but no other restrictions were placed on the number or type of previous lines of therapy. The study started accrual in February 2017, and enrolled globally, including in countries lacking access to immunotherapy. As a result, 68 % of patients did not receive a first-line checkpoint inhibitor (CPI) before entering the trial.

In oncology trials, the control arm can offer a limited number of options, often referred to as “physician's” or “investigator's” choice treatment [3]. The primary concern with such restricted choices arises when they exclude a significant treatment alternative from the available options. In the context of LUNAR, one might contend that patients without previous exposure to immunotherapy could then receive a CPI within the trial, simply because the “standard therapy” arm proposed this option. LUNAR imposed another limitation: the “standard therapy choice” was limited by drug availability [3]. Therefore, patients in countries lacking access to immunotherapy had no “choice” besides docetaxel. As such, 41 % of patients received docetaxel in LUNAR without having received prior CPI.

Another concern is that a subgroup analysis in LUNAR suggested that the survival benefit was limited to patients receiving concurrent CPI and was not observed in the “docetaxel” subgroup. We have already noted that not all patients in the “docetaxel” group had previously received CPI, which is a separate concern. However, this subgroup was closer to the standard-of-care, with 59 % of patients having received prior CPI. Therefore, it is even more questionable to adopt a treatment that did not show a benefit in the subgroup of patients who had the most exposure to first-line immunotherapy, which is the standard-of-care.

Given the efficacy of targeted therapy in patients harboring molecular alterations like EGFR mutations or ALK rearrangements, limited access to such therapy may also impact survival. Such data were not collected and are lacking in LUNAR [2].

Ultimately, because 1) two-thirds of patients did not receive prior immunotherapy, 2) 41 % of patients received docetaxel in LUNAR with no prior exposure to immunotherapy, 3) no benefit was seen in the subgroup most closely approaching the current standard of care, and 4) some patients may have harbored actionable mutations, and did not receive targeted therapy, the applicability of LUNAR to US and European practice may be questioned.

## A local treatment that extends survival: bioplausibility is lacking

In advanced cancer, the likelihood of local treatments like TTFs significantly prolonging survival is limited. Advanced cancer often involves widespread metastases, requiring systemic rather than localized intervention. TTFs, while innovative in targeting cancer cells through electric fields at a specific site, don't address cancer's systemic spread. Therefore, while TTFs might offer some benefits, their impact on overall survival in advanced cancer stages seems, prima facie, unlikely.

## Survival analysis: an apparent benefit, yet many unanswered questions

During LUNAR, it was decided to reduce the sample size after the Data Monitoring Committee (DMC) requested an unplanned interim analysis due to slow accrual. However, the DMC may have departed from pre-specified statistical rules. Unfortunately, even when asked for clarification [4]. the DMC analysis remain unshared, and no explanation has been provided [5].

In the absence of prespecified and stated reasons to alter sample size, concern arises that the decision was influenced by a chance deviation in data that favored the intervention. It may be tempting to “quit while you are ahead”, and seek to curtail a trial trending in a favorable direction.

Another key interrogation is related to rates and reasons for censoring. We noticed higher proportion of censored patients in the TTFs arm, which may signal that differential and informative censoring occurred in the survival analysis. Here, withdrawals should minimally affect rates of censoring, simply because patients’ survival status can be tracked even after they quit a trial. Yet, after the investigators shared reasons for censoring [5], this confirmed our initial concerns: after excluding patients censored because of data cut-off, the TTFs arm showed a 10 percentage-point higher rate of censoring, largely due to withdrawal of consent.

It is likely that frailer patients, which are also those who are more likely to experience an event (death), also drop-out more rapidly when facing toxicity. When this happens, the group with more toxicities will retain better prognosis patients, artificially favoring this group [6]. As serious adverse events occurred in 53 % of patients receiving TTFs and 38 % in the control group, informative censoring could have occurred, artificially boosting a survival gain which may not have been similar were those patients not censored.

We explored how this could have distorted the survival results presented in LUNAR by reconstructing individual patient data (IPD) based on the published Kaplan-Meier curves. When we hypothesized that half of the excess censored patients in the TTFs arm at 12 months would have presented an event instead of being censored, the survival advantage in LUNAR loses its statistical significance [7].

The second concern, in short, is that because of sample size modification, and because informative censoring may have occurred, the survival gain reported in the LUNAR trial is questionable.

## Open-label design, and the lack of a sham-control

While the open-label design may pose certain biases, a significant concern emerges from the additional care associated with TTFs. Patients and their family in the TTFs arm not only received the therapy but also benefited from continuous 24/7 support, frequent home-based interactions for array changes every three days, and the convenience of direct home deliveries for the device. To what extent was the observed survival advantage influenced by the therapy itself versus the byproduct of increased vigilance? This could explain why TTFs showed benefit in LUNAR, and previously in glioblastoma [8], both conditions with a poor prognosis, but not in ovarian cancer, a less aggressive condition [9].

Drawing a parallel, early introduction of palliative care in advanced or metastatic lung cancer increased survival by 2.7 months [10]. In the case of TTFs, the ideal study design would involve a sham device, ensuring that both groups receive equal attention, thereby isolating the true impact of TTFs on survival. A sham procedure is the equivalent of placebo for procedures like surgery. When medical practices are implemented, and later reversed based on stronger evidence, like this was the case for vertebroplasty in those settings, this is called a “reversal” [11]. It is possible that an adequately powered, sham-controlled trial could lead to such a reversal in the case of TTFs.

Our third conclusion is that without a sham-controlled trial, the true effect of TTFs is impossible to isolate.

## Funding

This project was funded by Arnold Ventures, LLC through a grant paid to the University of California, San Francisco.

## Conclusion

Current data of TTFs in NSCLC have minimal impact in practice, because patients do not mirror those enrolled in LUNAR. Many factors may have boosted the survival gain: higher rate of censoring in the TTFs arm, reduced sample size with the risk of fluke results, and absence of sham-control. A sham-controlled trial would be key in answering whether TTFs are beneficial. In the meantime, regulators should lean toward protecting poor prognosis patients who will naturally seek for more options. Those should not be exposed to procedures with significant toxicities before reaching sound confidence that they provide a clinical benefit.

## CRediT authorship contribution statement

**Timothée Olivier:** Writing – review & editing, Writing – original draft, Validation, Software, Investigation, Conceptualization. **Vinay Prasad:** Writing – review & editing, Supervision, Methodology.

## Declaration of competing interest

Dr Vinay Prasad reported receiving research funding from Arnold Ventures LLC through a grant made to UCSF; royalties for books and writing from Johns Hopkins Press, MedPage, and the Free Press; and consulting fees from UnitedHealthcare and OptumRX. He also reported receiving revenue from Patreon, YouTube, and Substack for the podcasts Plenary Session, VPZD, and Sensible Medicine; for the newsletters Sensible Medicine, The Drug Development Letter, and VP's Observations and Thoughts; and for the YouTube channel Vinay Prasad MD MPH. Dr Timothée Olivier has no conflicts of interest to declare.
